# When health research feels relevant: a data report on perceived research applicability and health behavior intentions

**DOI:** 10.3389/fpsyg.2026.1803355

**Published:** 2026-07-14

**Authors:** Amy Wang, Elsie Wong, Antonio L. Freitas

**Affiliations:** Department of Psychology, Stony Brook University, Stony Brook, NY, United States

**Keywords:** diversity, health behavior, health communication, scale development, trust in science

## 1 Introduction

Marginalized populations face elevated risks of developing health conditions across multiple domains. For example, differences in race, gender, and socioeconomic status (SES) all relate to incidence and outcomes for cancer (Islami et al., 2022), cardiovascular disease (Connelly et al., 2021; Javed et al., 2022), and mental health (Campbell et al., 2021; Kivimäki et al., 2020; McKnight-Eily et al., 2021). One contributor to health disparities is differences in health behaviors. Past research has identified racial, gender, and socioeconomic disparities in behaviors such as physical activity, smoking, and alcohol consumption (Abdel-Rahman, 2021; Bolen et al., 2000; Pampel et al., 2010).

Despite these disparities, minoritized identities are often understudied in health research. For decades, US federal policy has declared it a priority to include women and underrepresented-racial/ethnic minorities in clinical trials research, but this goal has not been met (Bibbins-Domingo et al., 2022). One study investigating clinical trials research in the U.S. found that 80% of participants reported being White, which is higher than the Census estimate of 72% White among the greater US population (Turner et al., 2022). This leaves underrepresented other racial/ethnic populations, including American Indian, Asian, and Latin American people, with Latin American populations showing the greatest disparity (Turner et al., 2022).

Basing conclusions on homogenous participant samples can make it difficult for scientists to interpret research results. Researchers may erroneously present their findings as if they apply to all people, even if diverse populations were not sampled (Simons et al., 2017). This can be potentially problematic in health research because different populations can show differences in disease presentation or treatment responses. One meta-analysis concerning adverse drug effects found that 27 out of 40 included studies identified differences by race or ethnicity (Baehr et al., 2015). In addition, 21% of new drugs showed differences in response based on patient race or ethnicity (Ramamoorthy et al., 2015). These findings demonstrate a need for scholars to be explicit about who exactly the target populations of their research are, because this information may influence how scientists form conclusions from research results. However, past research has not examined how lay people think about the target populations of research results, and importantly, whether they consider themselves to be within the target population.

This data report presents two datasets that administered a newly created questionnaire named the *perceived research applicability* (PRA) measure. The PRA measure is intended to assess the extent to which people perceive research results as being personally applicable to oneself. The measure consists of two factors. The *applicability* factor measures people's perceptions about the feasibility of implementing a research-recommended behavior in their own lives. The *transferability* factor measures the extent that people perceive a recommended behavior as being effective for themselves personally. The PRA measure was administrated in a repeated-measures format, meaning it can detect differences within-person (i.e., how the same individual varies across different health situations) and between-persons (i.e., how different individuals systematically differ on average). This is critical because within- and between-person relationships are statistically independent, meaning they can be different in directionality, form, and size (Dalal et al., 2014; Rohrer and Murayama, 2023).

These two datasets also include data related to health behaviors, trust in science (Dataset 2 only), and participant demographics. These datasets can be useful for researchers to examine how the PRA measure relates to these variables. The measures and stimuli developed for these datasets are publicly available, which may help future researchers design related studies. However, the PRA measure is still in its preliminary stages of development and requires further psychometric validation.

## 2 Data availability

Both datasets are publicly available on ResearchBox through the link: https://researchbox.org/4673&PEER_REVIEW_passcode=RQMBDA. For each dataset, we present the Qualtrics survey materials, deidentified raw data (in CSV format) with associated codebooks describing variable names, Supplemental materials (including Excel files describing the factor loadings from exploratory factor analysis), and two R files containing annotated code for data analysis. One *R* file contains the main analyses, which includes data cleaning (dropping participants who failed attention checks, reverse-coding measure items), visualizing scree plots, exploratory factor analysis, descriptive statistics, multilevel modeling, and both pre-registered and exploratory analyses with demographic traits. The other R file contains code related to calculating reliability of the PRA measure.

## 3 Dataset 1

In the procedures that produced this dataset, participants were presented with brief descriptions of research-based health recommendations, and they answered questions about each recommendation. This study was pre-registered using the As Predicted platform (https://aspredicted.org/r9r4-drmj.pdf).

### 3.1 Materials and methods

#### 3.1.1 Procedure

We administered an online survey using the platform Qualtrics. The survey consisted of eight blocks, with each block corresponding to one of the eight health recommendations, presented in a random order. In each block, participants first reported how frequently they engaged in the recommended behavior. Then, participants were presented with a short description of a research study that recommended performing a particular health behavior. Participants then rated their intentions to increase their performance of the behavior. Afterwards, they completed the 28-item PRA measure. Lastly, participants completed a demographic questionnaire.

#### 3.1.2 Stimuli

##### 3.1.2.1 Health recommendations

We presented participants with eight health recommendations related to preventing cardiovascular disease. Each health recommendation consisted of a two-sentence description of a research study that found that doing a certain behavior could reduce cardiovascular disease risk. These behaviors included flossing, avoiding working at night, avoiding noisy environments, listening to classical music, eating spicy foods, avoiding naps, volunteering, and watching less television. We selected these behaviors because we wanted recommendations that would not seem overly obvious to participants to avoid ceiling effects on intention.

#### 3.1.3 Measures

##### 3.1.3.1 Repeated measures

***Frequency***. The baseline frequency of doing a particular behavior was assessed prior to showing participants the health recommendation. This item read: “Please rate the extent to which you agree with the following statement: [Doing X behavior] is something that I do frequently.” Participants rated their agreement with this statement using a 7-point Likert scale, ranging from 1 (*Strongly disagree*) to 7 (*Strongly agree*). The frequency for four of the health recommendations were reverse-coded for analysis. This is because for these items, participants were asked to report their frequency of doing the behavior, whereas the health recommendation suggested participants to *avoid* doing that behavior.

***Intention***. Intention to follow the recommended behavior was measured immediately after presenting participants the health recommendation. The item stated: “After reading about this research study, I intend to [follow X recommendation] more.” Participants were then instructed to rate their agreement with this statement using a 7-point Likert scale, with 1 being *Strongly disagree* and 7 being *Strongly agree*.

***Perceived Research Applicability***. In this first study, PRA was measured using a newly developed 28-item scale, presented in a random order. The scale included a mixture of forward-coded and reverse-coded items. Each item was scored using a 7-point Likert scale, ranging from 1 (*Strongly disagree*) to 7 (*Strongly agree)*. Scores were calculated for each participant by reverse-coding necessary items and then averaging together item scores for each of the eight health recommendations. Because this was the first time this measure had ever been used, the reliability of the scale had not yet been assessed.

##### 3.1.3.2 Non-repeated measures

***Demographic Questionnaire***. After completing all other measures, participants filled out a demographic questionnaire, which included items related to age, gender, race/ethnicity, SES, sexual orientation, and disability status.

#### 3.1.4 Participants

We recruited 300 undergraduate students from a large northeastern U.S. university to participate in this study. We chose to recruit *N* = 300 participants; this is often considered the minimum recommended number of participants necessary for scale development (Carpenter, 2018; Clark and Watson, 1995) and is roughly aligned with a rule of thumb to include at least ten participants per item in the scale (Morgado et al., 2017). Students were recruited from the university's Department of Psychology subject pool and participated in exchange for course credit. Participants were ineligible if they were non-English-speaking or under 17 years old. Enrolled participants' data were excluded from analysis if they failed more than one of four attention check questions. This left 253 participants remaining for analysis, and any responses with partial missing data were retained. Demographic characteristics of the participant sample are described in Table 1.

**Table 1:** Participant demographics.

Sample Characteristics: *n* (% of total sample)	*Dataset 1 (N = 253)*	*Dataset 2 (N = 329)*
Age (in years)	*M* = 20.1 (*SD* = 2.3)	*M* = 19.6 (*SD* = 2.3)
Gender^*^		
Man	89 (35.2%)	119 (36.2%)
		
Woman	154 (60.9%)	202 (61.4%)
		
Nonbinary	1 (0.4%)	6 (1.8%)
		
Transgender	3 (1.2%)	5 (1.5%)
		
Did not answer	9 (3.6%)	4 (1.2%)
**Race and ethnicity** ^*^		
American Indian or Alaska Native	0 (0.0%)	2 (0.6%)
		
Asian	121 (47.8%)	176 (53.5%)
		
Black or African American	22 (8.7%)	23 (7.0%)
		
Hispanic, Latino, or Spanish Origin	44 (17.3%)	56 (17.0%)
		
Middle Eastern or North African	5 (2.0%)	7 (2.1%)
		
Native Hawaiian or Pacific Islander	1 (0.4%)	0 (0.0%)
		
White	88 (34.8%)	93 (28.3%)
		
White alone	66 (26.1%)	75 (22.8%)
		
Another race or ethnicity not listed	5 (2.0%)	7 (2.1%)
		
Did not answer	3 (1.2%)	5 (1.5%)
Socioeconomic status		
Childhood class		
Working class	62 (24.5%)	87 (26.4%)
		
Lower middle class	86 (34.0%)	108 (32.8%)
		
Upper middle class	75 (29.6%)	102 (31.0%)
		
Upper class	4 (1.6%)	3 (0.9%)
		
Did not answer	26 (10.3%)	29 (8.8%)
Current class		
Working class	60 (23.7%)	77 (23.4%)
		
Lower middle class	70 (27.7%)	88 (26.7%)
		
Upper middle class	88 (34.7%)	125 (38.0%)
		
Upper class	10 (4.0%)	7 (2.1%)
		
Did not answer	25 (9.9%)	32 (9.7%)
Sexual identity		
LGBTQ+ identifying	37 (14.6%)	65 (19.8%)
Straight/heterosexual	207 (81.8)	248 (75.4%)
Did not answer	9 (3.6%)	16 (4.9%)
Disability status		
Identifying with a disability	42 (16.6%)	62 (18.8%)
No disability or impairment	191 (75.5%)	244 (74.2%)
Did not answer	20 (7.9%)	23 (7.0%)

^*^Participants were permitted to select multiple options for gender and race/ethnicity. For this reason, the associated percentages sum to over 100%.

### 3.2 Factor structure of the PRA measure

The original PRA item pool was developed to assess three conceptual domains of applicability, transferability, and identity, and the corresponding item names in the Supplemental materials reflect these initial content categories. To assess empirical support for this hypothesized factor structure, we generated scree plots of the PRA scores for each of the eight recommendations. Most scree plots showed an elbow around the second factor, suggesting that the PRA measure can be characterized by two rather than three factors.

Next, we performed exploratory factor analysis (EFA) separately for each of the eight health recommendations. We chose to use EFA rather than confirmatory factor analysis because the measure was newly created and lacked empirical support, so an exploratory approach was appropriate. To do this, we used the function *factanal ()* in *R*, using a maximum likelihood extraction method, orthogonal rotation, and two factors. As a general trend, items that aligned with the transferability concept tended to cluster together, and they usually formed the factor that explained the greater amount of variance. Additionally, items more aligned with the applicability concept also tended to cluster together, forming the second factor. However, there was not consistent evidence for a distinct identity factor across recommendation domains, so we decided to drop the hypothesized identity subscale from future iterations of the measure. A complete table of item loadings is available as Supplemental materials in the ResearchBox repository.

We used the EFA results to inform our decisions about which items to cut from future versions of the measure. We removed the items that showed the least consistent factor loadings across the eight health recommendations, attempting to create a two-subscale measure with the same number of items per subscale. This process was exploratory, and we did not follow any pre-defined numeral cutoff criteria for our decisions. We were left with 16 remaining items, composed of two eight-item subscales tentatively interpreted to represent transferability and applicability.

To calculate reliability, we used McDonald's omega for within-person reliability and Cronbach's alpha for between-person reliability. The reduced 16-item scale was highly reliable both within-persons (ω = 0.91) and between-persons (α = 0.94). The 8-item applicability subscale was also reliable (within-persons: ω = 0.86, between-persons: α = 0.91), as was the 8-item transferability subscale (within-persons: ω = 0.83, between-persons: α = 0.94).

## 4 Dataset 2

Because of the exploratory nature of the scale refinement conducted in Dataset 1, a replication of this work is warranted. Dataset 2 presents a replication and extension of the work conducted in Dataset 1. This study was pre-registered on Open Science Framework (https://osf.io/twr7f/?view_only=415e0a0d25f349deb3f4f47d8cf35aba).

### 4.1 Materials and methods

#### 4.1.1 Procedure

The procedure for Dataset 2 largely replicated that for Dataset 1. Key changes included the addition of health recommendations related to anxiety, the use of a reduced PRA scale, and the addition of new non-repeated measures.

#### 4.1.2 Stimuli

##### 4.1.2.1 Health recommendations

We presented participants with eight health recommendations, four related to cardiovascular disease (flossing, avoiding noisy environments, listening to classical music, and volunteering) and four related to anxiety (avoiding skipping meals, sleeping earlier, exercising in green spaces, and eating more probiotic foods). The new health recommendations were added to explore whether the PRA measure could be applicable to other health domains.

#### 4.1.3 Measures

##### 4.1.3.1 Repeated measures

Dataset 2 used the same frequency and intention measures as Dataset 1. In this dataset, we used a modified version of the PRA scale, consisting of a 16-item scale with two subscales, applicability (eight items) and transferability (eight items).

##### 4.1.3.2 Non-repeated measures

***Trust in Science and Scientists Inventory***. Trust in science was measured using the Trust in Science and Scientists Inventory, which was originally developed as a 21-item measure by Nadelson et al. (2014). We used a shortened version of the measure, as described by Plohl and Musil (2021). The shortened version consists of 14-items and has high reliability (α = 0.81; Plohl and Musil, 2021). Each item is scored on a 5-point Likert scale, with 1 indicating *Strongly disagree* and 5 indicating *Strongly agree*.

***Fact-Checking Behavior***. Fact-checking behavior was measured using a 7-item scale developed by Stefanita et al. (2018), which asks participants to rate how often they engage in fact-checking techniques when consuming the news. Items were measured on a 7-point Likert scale ranging from 1 (*Strongly disagree*) to 7 (*Strongly agree*).

#### 4.1.4 Participants

A total of 363 undergraduate students participated in this study, following the same eligibility criteria as in Dataset 1. Participants were excluded if they failed more than one of four attention check questions, leaving 329 participants remaining for analysis. Sample demographics of Dataset 2 are summarized in Table 1.

### 4.2 Factor structure of the PRA measure

Scree plots from Dataset 2 largely showed an elbow around the second factor, suggesting that PRA is best characterized by two factors. Next, we conducted two-factor EFA using similar parameters as in Dataset 1. Items from the applicability subscale generally clustered into one factor, whereas items from the transferability subscale generally clustered into a separate factor.

Using EFA, we chose eight items to cut from future versions of the measure, leaving eight remaining items, comprised of a four-item applicability subscale and four-item transferability subscale. The overall eight-item scale was also highly reliable within-persons (ω = 0.87) and between-persons (α = 0.88). Additionally, the shortened applicability subscale was found to be highly reliable both within (ω = 0.81) and between-persons (α = 0.88), as was the transferability subscale (within-persons: ω = 0.81; between-persons: α = 0.86).

## 5 Data description

We assessed descriptive statistics for both datasets. In Dataset 1, the average score for the full PRA measure was *M* = 4.62 (*SD* = 0.57), the applicability subscale was *M* = 4.97 (*SD* = 0.64), and the transferability subscale was *M* = 4.44 (*SD* = 0.74). Average frequency was *M* = 3.65 (*SD* = 0.79), and intention was *M* = 4.31 (*SD* = 1.00). In Dataset 2, the average PRA was *M* = 4.89 (*SD* = 0.61), applicability was *M* = 5.12 (*SD* = 0.72), and transferability was *M* = 4.68 (*SD* = 0.74). Average frequency was *M* = 3.40 (*SD* = 0.76), and intention was *M* = 4.49 (*SD* = 1.05).

To examine the separate within- and between-person characteristics of the PRA measure, we used multilevel modeling. First, we fitted empty models with PRA as the outcome, and in both datasets, we found similarly low intraclass correlation coefficient (ICC) values (Dataset 1: ICC = 0.28, Dataset 2: ICC = 0.26). This indicates that 28% (in Dataset 1) or 26% (in Dataset 2) of the variance in PRA was due to between-person differences. The remaining variance was likely attributed to within-person differences and error.

### 5.1 Within-person characteristics

Next, we fitted multilevel models using intention as the outcome variable and with PRA and baseline frequency as predictor variables. In both datasets, the models showed an association between higher within-person PRA and higher intention when frequency is held constant (Dataset 1: *b* = 1.02, *SE* = 0.04, *p* < 0.0001; Dataset 2: *b* = 0.91, *SE* = 0.03, *p* < 0.0001).

### 5.2 Between-person characteristics

The same multilevel models also showed a relationship between higher between-person PRA and higher intention when frequency is held constant (Dataset 1: *b* = 0.41, *SE* = 0.10, *p* = 0.0001; Dataset 2: *b* = 0.83, *SE* = 0.07, *p* < 0.0001).

In addition, we used Pearson correlations to examine between-person relationships between PRA, intentions, race, SES, and trust in science (with the latter variable included in Dataset 2 only), as reported in Table 2. Notably, the relationships between race, SES, and PRA were inconsistent between Datasets 1 and 2. Dataset 1 found no racial differences in PRA (*p* = 0.132), whereas Dataset 2 found that White participants scored higher in PRA than non-White/multiracial participants (*p* = 0.023). In contrast, Dataset 1 found that higher SES participants scored higher in PRA (*p* = 0.036), whereas that association was not significant in Dataset 2 (*p* = 0.463).

**Table 2:** Correlation coefficients and 95% confidence intervals for between-person analyses.

	PRA	Intention	Race	SES	Trust in Science
1. PRA	–	0.28^**^ [0.16, 0.40]	0.10 [−0.03, 0.23]	0.14^*^ [0.01, 0.27]	N/A
2. Intention	0.56^**^ [0.48, 0.63]	–	−0.11 [−0.24, 0.02]	0.07 [−0.06, 0.20]	N/A
3. Race	0.13^*^ [0.02, 0.24]	0.11 [0.00, 0.22]	–	0.24^**^ [0.12, 0.36]	N/A
4. SES	0.04 [−0.07, 0.16]	0.02 [−0.10, 0.13]	0.11 [−0.01, 0.22]	–	N/A
5. Trust in science	0.34^**^ [0.23, 0.43]	0.02 [−0.10, 0.13]	0.16^**^ [0.04, 0.27]	0.06 [−0.06, 0.17]	–

Values above the diagonal indicate data from Dataset 1, whereas values below the diagonal are from Dataset 2. ^*^ indicates *p* < 0.05, and ^**^ indicates *p* < 0.01. Race was operationalized as non-White/multiracial = 0 and White = 1. Trust in science was assessed in Dataset 2 only.

## 6 Limitations

We present two datasets detailing the preliminary development of a novel PRA measure. These data show high reliability for the PRA measure, but more research is needed to assess the validity of this measure, including its convergent, divergent, and criterion validity. The process used to refine and delete scale items was exploratory in nature. Future research is needed to replicate these exploratory results, including using confirmatory factor analysis to confirm the factor structure of the measure.

One major limitation is that the datasets recruited undergraduate psychology student participants, who lack diversity in key aspects such as age and education level. This lack of diversity can make it difficult to evaluate how the PRA measure may relate to demographic characteristics. Additionally, both datasets had relatively high rates of data exclusion due to failed attention checks. This suggests that participants may not have been very engaged in the study procedures, possibly due to the long survey length and online nature of the studies. Future research using the PRA measure should recruit more diverse participant samples and use the shortened version of the measure to mitigate inattentiveness during the survey.

## Data Availability

The datasets presented in this study can be found in online repositories. The names of the repository/repositories and accession number(s) can be found in the article/Supplementary material.
